# Bioconversion of lignocellulosic waste to bioethanol by *Trichoderma* and yeast fermentation

**DOI:** 10.1007/s13205-013-0179-4

**Published:** 2013-10-24

**Authors:** K. Saravanakumar, K. Kathiresan

**Affiliations:** Faculty of Marine Sciences, Centre of Advanced Study in Marine Biology, Annamalai University, Parangipettai, 608502 Tamil Nadu India

**Keywords:** Bioethanol, Marine yeasts, *Trichoderma*, Lignocellulosic waste, Mangroves

## Abstract

The present work aimed at producing bioethanol using lignocellulosic waste sawdust and marine yeast fermentation. Lignocellulosic waste materials were converted into monosugars through acid hydrolysis and finally treated with cellulase enzyme derived from *Trichoderma*/*Hypocrea*. To enhance the conversion of the glucose from sawdust, the experimental conditions were statistically optimized. The efficient conversion of sawdust to glucose of 78.56 % was achieved under the conditions of pH 6.19, temperature 29 °C, cellulase enzyme (8.16 IU ml^−1^) and sawdust (7.95 g l^−1^). The lignocellulosic waste-sawdust hydrolysis was used as the carbon source for the production of bioethanol. Bioethanol production of 85.6 % was achieved (55.2 g l^−1^) under the optimized conditions of temperature of 36.5 °C, incubation time of 102 h and enzyme-treated sawdust of 45.14 ml l^−1^ and agitation of 330 rpm. This work achieved maximum bioethanol production using *H.estonica* and *S.cerevisiae* fermentation.

## Introduction

Fuel deficiency is a global issue due to exhaustion of fossil fuel and growing climate change (EC 2003; Wingren et al. 2003; Li 2003; Qureshi et al. 2006; Sveinsdottir et al. 2009). In order to overcome this issue, different types of techniques have been invented for the possible conversion of cellulosic waste materials into glucose for the ethanol production, as an alternative way for fuel conservation (Sun and Cheng 2002). The bioconversion of lignocellulosic materials (i.e., agricultural residues, woods, and residues from pulp and paper industries, solid wastes) to bioethanol produces high yield of glucose after hydrolysis (McMillan 1994). The utilization of the lignocellulosic materials for the conversion of the biofuel involves only low cost (Li et al. 2007).

Lignocellulose, the most abundant organic matter in the Earth, can be utilized to produce various renewable fuels and chemicals (Lau et al. 2010). For the digestion of these materials into glucose, many methods have been used such as thermal pretreatments, chemical pretreatments, biological pretreatments and enzymatic pretreatments (Meinita et al. 2012). Cellulolytic and hemicellulolytic enzymes are able to increase monosugars from the digestion of lignocellulose (Pakarinen et al. 2011). For this purpose, fungi in particular *Trichoderma* species that can produce cellulolytic enzymes are employed (Aro et al. 2005; Rodriguez Gomez et al. 2012). In general, yeasts are potential microorganisms for the production of bioethanol by sugar fermentation (Kathiresan et al. 2011; Senthilraja et al. 2011). However, most of the studies are restricted to microbes of terrestrial origin, but not of marine origin. Hence, the present work was undertaken for the conversion of sawdust (lignocellulosic wastes) to sugars for fuel fermentation using cellulolytic enzymes of the mangrove-derived *Trichoderma* and further conversion of sugars to bioethanol using the mangrove-derived yeast strain of *Saccharomycescerevisiae*. Based on the data, the culture conditions were optimized statistically for the enzyme hydrolysis process and bioethanol fermentation.

## Materials and methods

### Microorganisms and maintenance

*Trichoderma* (*Hypocrea*) species (*Trichodermaestonicum/H.estonica* SKS1 JQ611722) and yeasts species (*Saccharomycescerevisiae* JN387604) were isolated from mangrove sediment, located in the south east coast of India using selective medium (Askew and Laing 1993) and yeast peptone agar medium (Fell 2005), respectively. The stock cultures were maintained at 4 °C on slants of potato dextrose agar for *H.estonica* and yeast peptone agar for *S.cerevisiae*.

### Substrate

Sawdust was obtained from local wood mills, passed through a 1.5-mm sieve to maintain uniform particle size, washed through distilled water to remove impurities present in it and finally dried at 60 °C overnight. The sawdust was pre-hydrolyzed using 0.8 % of phosphoric acid by the method of Kathiresan et al. (2011).

### Cellulase enzyme production by *Hypocreaestonica*

Spore suspension of *Hypocrea estonica* SKS1 (JQ611722) was used as microbial inoculum for the fermentation. It was prepared by culturing *H.estonica* in potato dextrose agar slant cultures at 30 °C for 7 days and spore suspension was washed through the Tween-80 water (0.02 % v/v); the suspension was then assessed as final spore count of 2.3 × 10^3^ CFU ml^−1^. Then 10 ml of spore suspension was inoculated into a 250-ml Erlenmeyer flask containing 100 ml glucose pre-cultured medium (%, w/v) (glucose, 3; yeast extract, 0.8; K_2_HPO_4_, 0.4; MgSO_4_·7H_2_O, 0.2; pH, 7.0) and was cultured at 30 °C, 200 rpm for 48 h in a rotary shaking incubator to prepare the mycelial suspension. At last, mycelial suspension was inoculated into a 250-ml Erlenmeyer flask containing the statistically optimized medium for cellulase production by *H.estonica* (Saravanakumar and Kathiresan 2013a) that consisted of 7.69 g l^−1^ of sawdust, 3.59 g l^−1^ of (NH_4_)_2_ SO_4_, under the temperature of 49 °C at pH of 8.8 with inoculum size of 6.0 % (v/w) and was cultivated at 30 °C for 120 h to produce cellulose.

## Determination of cellulase enzyme activity

5 ml of the fermented residues was suspended in 150 ml distilled water and shaken at 120 rpm for 2 h. Then the filtrate was centrifuged (10,000 rpm) using a high speed centrifuge for 15 min and the supernatant was used for enzyme assay using the dinitrosalicylic acid (DNS) method (Miller 1959).

## Enzymatic hydrolysis

Enzymatic hydrolysis was carried out in 125 ml screw-capped Erlenmeyer flasks. Diluted acid-treated sawdust powder was suspended in distilled water in the flasks. Water was added during the pretreatment in such a way so as to maintain the substrate concentration at 15 % (w/v) after the addition of the crude cellulase enzyme derived from *H.estonica*. Hydrolysis was performed at 50 °C for 24 h at 120 rpm in an incubator shaker. 1 ml sample was periodically removed from each flask (0, 2, 8, 18, 24, 72, 120, and 168 h). Each sample was centrifuged for 10 min at 13,500*g*, and 500 μl supernatant was then removed and placed into a 1.5-ml Eppendorf tube containing the stop buffer (512 mM Na_2_CO_3_ and 288 mM NaHCO_3_; pH 10.0) (Sandhu et al. 2012). The buffered samples were stored at 4 °C for subsequent glucose measurement; commercial glucose was used as the standard for the percentage calculation.

## Bioethanol production by *S.cerevisiae*

Bioethanol production experiments were carried out according to the experimental setups derived from the center composite design. The enzyme-treated sawdust hydrolysis was used as the carbon source. The percentage of the bioethanol was calculated according to the method of Saravanakumar et al. (2013b).

## Determination of bioethanol using gas chromatography

Concentration of bioethanol in the distillate of culture filtrate was estimated using a Hewlett Packard 5890 Series II gas chromatography with chromosorb 105 column and nitrogen as a carrier gas. The temperature of the injection port, oven and detection port was 250, 120, and 250 °C, respectively. For the analysis, 1 μl of liquid samples was injected into gas chromatography. The bioethanol concentration was determined using bioethanol standard plot and is expressed in percentage with help of instrumental default standard value of 65 g l^−1^ which is equivalent to 100 % (Saravanakumar and Kathiresan 2013a, 2013b).

## Optimization of the enzyme hydrolysis process

Optimization of the environmental conditions for the maximum hydrolysis process of the sawdust to sugar using *H.estonica*-derived cellulase was studied using 30 runs of statistical model assessed from central composite design of response surface methodology. In this experiment, important factors such as pH (6–8), temperature (20–50 °C) and cellulase concentration (2–10 IU ml^−1^) on glucose conversion were studied.

1 $$Y\;=\;\beta_{0}+\Sigma_{i}\beta_{i}X_{i}+\Sigma_{i}\beta_{ii}X_{i}^{2}+\Sigma_{ij}\beta_{ij}X_{i}X_{j}$$

where *Y* is the predicted response (glucose), *X*_*i*_, *X*_*j*_ the independent variables, *β*_0_ the offset term, *β*_*i*_ the *i*th linear coefficient, *β*_*ii*_ the *i*th quadratic coefficient and *β*_*ij*_ is the *ij*th interaction coefficient. The experimental design in coded and uncoded value is presented in Table 1. In this study, the independent variables are coded as *X*_1_, *X*_2_, and *X*_3_, Thus, the second-order polynomial equation can be presented as follows:

2 $$\begin{array}{c} Y\;=\;\beta_{0}+\;\beta_{1}X_{1}+\;\beta_{2}X_{2}+\;\beta_{3}X_{3}+\;\beta_{4}X_{4}+\;\beta_{11}X_{1}^{2}+\;\beta_{22}X_{2}^{2}+\;\beta_{33}X_{3}^{2}+\;\beta_{44}X_{4}^{2}+\;\beta_{12}X_{1}X_{2}\\ +\beta_{13}X_{1}X_{3}+\;\beta_{14}X_{1}X_{4}+\;\beta_{23}X_{2}X_{3}+\;\beta_{24}X_{2}X_{4}+\;\beta_{34}X_{3}X_{4} \end{array}$$

**Table 1:** Center composite design of response surface methodology for the glucose production of experimental and predicted responses

Std	(A) pH	(B) Temperature (°C)	(C) Cellulase concentration (IU ml^−1^)	(D) Sawdust (mg l^−1^)	Glucose (%)	
					Experimental	Predicted
1	6	20	2	2	65.26	63.13
2	8	20	2	2	52.26	44.33
3	6	50	2	2	42.56	34.68
4	8	50	2	2	32.26	31.08
5	6	20	10	2	53.26	52.91
6	8	20	10	2	41.23	31.96
7	6	50	10	2	23.25	19.11
8	8	50	10	2	15.26	13.36
9	6	20	2	10	45.65	52.32
10	8	20	2	10	36.56	31.85
11	6	50	2	10	35.62	36.04
12	8	50	2	10	25.65	30.77
13	6	20	10	10	78.56	70.89
14	8	20	10	10	35.62	48.27
15	6	50	10	10	36.56	49.26
16	8	50	10	10	48.56	41.84
17	5	35	6	6	68.56	67.71
18	9	35	6	6	36.56	41.49
19	7	5	6	6	45.56	49.89
20	7	65	6	6	15.26	15.01
21	7	35	2	6	35.26	39.03
22	7	35	14	6	39.56	39.87
23	7	35	6	2	12.25	27.61
24	7	35	6	14	56.56	45.28
25	7	35	6	6	65.23	65.23
26	7	35	6	6	65.23	65.23
27	7	35	6	6	65.23	65.23
28	7	35	6	6	65.23	65.23
29	7	35	6	6	65.23	65.23
30	7	35	6	6	65.23	65.23

### Statistical optimization of bioethanol production

In this experiment, *S.cerevisiae* (JN387604) was selected for optimization. The individual and interaction effects of temperature (°C), incubation time (0–120 h), and enzyme-treated sawdust (10–50 ml l^−1^) and agitation (100–500) on bioethanol production were carried out. The maximum yield of bioethanol production was tested using 30 experimental setups derived from a statistical model-central composite design of response surface methodology. The coded values of the fermentation factors were determined by the following equation:

3 $$Y\;=\;\beta_{0}+\;\Sigma_{i}\beta_{i}X_{i}+\;\Sigma_{i}\beta_{ii}X_{i}^{2}+\;\Sigma_{ij}\beta_{ij}X_{i}X_{j}$$

where *Y* is the predicted response, *X*_*i*_, *X*_*j*_ the independent variables, *β*_0_ the offset term, *β*_*i*_ the *i*th linear coefficient, *β*_*ii*_ the *i*th quadratic coefficient and *β*_*ij*_ is the *ij*th interaction coefficient. The experimental design in coded and uncoded value is presented in Table 2. In this study, the independent variables are coded as *X*_1_, *X*_2_, *X*_3_ and *X*_4_. Thus, the second-order polynomial equation can be presented as follows:

4 $$\begin{array}{c} Y\;=\;\beta_{0}+\;\beta_{1}X_{1}+\;\beta_{2}X_{2}+\;\beta_{3}X_{3}+\;\beta_{4}X_{4}+\;\beta_{11}X_{1}^{2}+\;\beta_{22}X_{2}^{2}+\;\beta_{33}X_{3}^{2}+\;\beta_{44}X_{4}^{2}+\;\beta_{12}X_{1}X_{2}\\ +\beta_{13}X_{1}X_{3}+\;\beta_{14}X_{1}X_{4}+\;\beta_{23}X_{2}X_{3}+\;\beta_{24}X_{2}X_{4}+\;\beta_{34}X_{3}X_{4} \end{array}$$

**Table 2:** Center composite design of response surface methodology for the bioethanol production of experimental and predicted response

Std	(A) Temperature (°C)	(B) Incubation time	(C) Enzyme-treated sawdust (ml l^−1^)	(D) Agitation (rpm)	Bioethanol yield (%)	
					Experimental	Predicted
1	0	0	10	100	0.00	4.71
2	50	0	10	100	0.00	9.51
3	0	120	10	100	15.69	19.99
4	50	120	10	100	75.56	82.40
5	0	0	50	100	0.00	4.24
6	50	0	50	100	0.00	8.58
7	0	120	50	100	15.65	19.09
8	50	120	50	100	85.65	81.04
9	0	0	10	500	0.00	21.21
10	50	0	10	500	0.00	18.69
11	0	120	10	500	12.26	25.81
12	50	120	10	500	68.56	80.92
13	0	0	50	500	0.00	15.28
14	50	0	50	500	0.00	12.30
15	0	120	50	500	12.36	19.45
16	50	120	50	500	56.69	74.10
17	25	60	30	300	15.58	−1.97
18	75	60	30	300	78.65	57.47
19	25	60	30	300	19.65	−8.25
20	25	180	30	300	79.65	68.82
21	25	60	10	300	75.26	49.04
22	25	60	70	300	54.26	41.75
23	25	60	30	100	15.26	16.12
24	25	60	30	700	65.26	25.67
25	25	60	30	300	75.60	75.60
26	25	60	30	300	75.60	75.60
27	25	60	30	300	75.60	75.60
28	25	60	30	300	75.60	75.60
29	25	60	30	300	75.60	75.60
30	25	60	30	300	75.60	75.60

A statistical program package Design Expert 8.0.6 was used for regression and model fit analysis of the data was obtained, and then estimated the coefficient of the regression equation, and analyzed the variance of selected factors and model significance.

## Results

### Effect of the cellulase enzyme on sawdust hydrolysis process

The interaction and individual effects of the experimental condition on hydrolysis process were studied by adopting the statistical design of the center composite model of 30 experimental setups and are presented in Table 1 along with the predicted and experimental responses of glucose production. The acceptability of the statistical model was tested by the quadratic model along with the contour error plot for the predicted and experimental responses and further model fitness tested by the normal standardized residual plot. The standard error value of 0.43 showed fitness for the acceptable model and *R*^2^ value of the model was 0.87 which also proved the 87 % of model fitness. The application of the response surface methodology based on the estimates of the parameters indicated an experimental relationship between the response and input variables with the glucose production expressed in the following quadratic mode:

5 $$\begin{array}{c} \mathrm{Glucose}\;\left(\%\right)=65.23-6.56X_{1}-8.72X_{2}+0.21X_{3}+4.42X_{4}+3.80X_{1}X_{2}-0.54X_{1}X_{3}-0.42X_{1}X_{4}\\ \;-1.34X_{2}X_{3}+3.04X_{2}X_{4}+7.20X_{3}X_{4}-2.66X_{1}^{2}-8.19X_{2}^{2}-6.44X_{3}^{2}-7.20X_{4}^{2} \end{array}$$

where *X*_1_ (pH), *X*_2_ (temperature), *X*_3_ (cellulase) and *X*_4_ (agitation) are independent variables. Significance of each coefficient is presented in Eq. (5), determined by the Student’s *t* test and *p* values (Montgomery 2001). The ANOVA results of the quadratic model for glucose production are given in Table 3. The value of *R*^2^ and adjusted *R*^2^ was close to 0.87 and it revealed a high correlation between the observed values and the predicted values. This means that regression model provides an excellent explanation of the relationship between the independent variables (factors) and the response (glucose production). The lack-of-fit term was non-significant as it was desired. The non-significant value of lack-of-fit observed (0.856) was more than probability of 0.05 and this revealed that the quadratic model was valid for the present study.

**Table 3:** Analysis of variance for the response of glucose production by *H. estonica*

Source	Sum of squares	*df*	Mean square	*F* value	*p* value prob > *F*
Model	8,011.377	14	572.2412	7.290321	0.0002***
(A) Ph	1,031.233	1	1,031.233	13.13785	0.0025**
(B) Temperature (°C)	1,824.922	1	1,824.922	23.2494	0.0002***
(C) Cellulase concentration (IU ml^−1^)	1.075267	1	1.075267	0.013699	0.9084^NS^
(D) Sawdust (mg l^−1^)	468.6968	1	468.6968	5.971171	0.0274*
AB	231.04	1	231.04	2.943437	0.1068^NS^
AC	4.6225	1	4.6225	0.05889	0.8115^NS^
AD	2.7889	1	2.7889	0.03553	0.8530^NS^
BC	28.6225	1	28.6225	0.364649	0.5550^NS^
BD	148.1089	1	148.1089	1.886899	0.1897^NS^
CD	828.8641	1	828.8641	10.55968	0.0054**
A^2^	193.6786	1	193.6786	2.467455	0.1371^NS^
B^2^	1,841.955	1	1,841.955	23.4664	0.0002***
C^2^	1,139.255	1	1,139.255	14.51405	0.0017**
D^2^	1,420.334	1	1,420.334	18.09498	0.0007***
Residual	1,177.399	15	78.49328		
Lack-of-fit	1,177.399	10	117.7399		0.856^NS^
Pure error	0	5	0		
Cor total	9,188.776	29			

*NS* not significant

*** *p* < 0.001; ** *p* < 0.01; * *p* < 0.05

In this case, the individual and interaction effects on the hydrolysis process were tested and the data are presented in Table 3. The factors of the *X*_1_, *X*_2_, *X*_4_, *X*_3_*X*_4_, *X*_2_^2^, *X*_3_^2^, *X*_4_^2^ were significant but other factors and their interaction effects were not significant on the glucose production (*p* < 0.05).

The optimization of the specific conditions for the glucose production was assessed. Statistically optimized conditions for the efficient sawdust hydrolysis process of glucose production were pH (6.19), temperature (29 °C), cellulase enzyme (8.16 IU ml^−1^) and sawdust (7.95 mg l^−1^). This hydrolysed glucose was used further for the alcohol production as the carbon source.

### Optimization of bioethanol production by *S.cerevisiae*

The interaction and individual effects of experimental conditions on bioethanol production process were studied by adopting the statistical design of the center composite for the bioethanol production. The acceptability of the statistical model was tested by the quadratic model along with the contour error plot for the predicted and experimental responses. The standard error value of the 0.43 showed fitness for the acceptable model and *R*^2^ value of the model of 0.81 also proved 81 % of model fitness. The application of the response surface methodology based on the estimates of the parameters indicated an experimental relationship between the response and input variables, which is expressed in the following quadratic model:

6 $$\begin{array}{c} \text{Bioethanol yield}\;\left(\%\right)=75.60+14.86X_{1}+19.27X_{2}-1.82X_{3}+2.39X_{4}+14.41X_{1}X_{2}-0.12X_{1}X_{3}-\\ 0.83X_{1}X_{4}-0.11X_{2}X_{3}-2.67X_{2}X_{4}-2.67X_{3}X_{4}-11.96X_{1}^{2}-11.33X_{2}^{2}-7.55X_{3}^{2}-13.68X_{4}^{2} \end{array}$$

where *X*_1_ (temperature), *X*_2_ (incubation time), *X*_3_ (enzyme-treated sawdust) and *X*_4_ (agitation) are independent variables. Significance of each coefficient is presented in Eq. (6), determined by the Student’s *t* test and *p* values (Montgomery 2001). The ANOVA results of the quadratic model for bioethanol production are given in Table 4. The value of *R*^2^ and adjusted *R*^2^ was close to 0.81 and it revealed a high correlation between the observed values and the predicted values. This means that regression model provides an excellent explanation of the relationship between the independent variables (factors) and the response (bioethanol production). The lack-of-fit term was non-significant. The non-significant value of lack-of-fit observed (0.52) was more than probability of 0.05 and this revealed that the quadratic model was valid for the present study.

**Table 4:** Analysis of variance for the response of bioethanol production by *S. cerevisiae*

Source	Sum of squares	*df*	Mean square	*F* value	*p* value prob > *F*
Model	28,097.22	14	2,006.944	4.824282	0.0023**
(A) Temperature (°C)	5,299.67	1	5,299.67	12.73932	0.0028**
(B) Incubation hours	8,909.677	1	8,909.677	21.41704	0.0003***
(C) Enzyme-treated sawdust (ml l^−1^)	79.64327	1	79.64327	0.191446	0.6680^NS^
(D) Agitation (rpm)	136.8993	1	136.8993	0.329078	0.5747^NS^
AB	3,320.641	1	3,320.641	7.982139	0.0128*
AC	0.2116	1	0.2116	0.000509	0.9823^NS^
AD	53.4361	1	53.4361	0.128449	0.7250^NS^
BC	0.1849	1	0.1849	0.000444	0.9835^NS^
BD	113.8489	1	113.8489	0.273669	0.6085^NS^
CD	29.75703	1	29.75703	0.07153	0.7928^NS^
A^2^	3,924.794	1	3,924.794	9.4344	0.0078**
B^2^	3,519.94	1	3,519.94	8.461214	0.0108*
C^2^	1,563.842	1	1,563.842	3.759156	0.0716^NS^
D^2^	5,129.922	1	5,129.922	12.33128	0.0031**
Residual	6,240.133	15	416.0088		
Lack-of-fit	6,240.133	10	624.0133		0.52^NS^
Pure error	0	5	0		
Cor total	34,337.35	29			

*NS* not significant

*** *p* < 0.001; ** *p* < 0.01; * *p* < 0.05

In this case, the individual and interaction effects on the hydrolysis process were tested and the data are presented in Table 4. The factors of the *X*_1_, *X*_2_, *X*_1_*X*_2_, *X*_1_^2^, *X*_2_^2^, *X*_3_^2^, *X*_4_^2^ were significant but other factors and their interaction effects were not significant on the bioethanol production (*p* < 0.05).

The optimization of the specific conditions for the bioethanol production was assessed. Statistically optimized conditions for the efficient bioethanol production process were temperature of 36.5 °C, incubation time of 102 h and enzyme-treated sawdust of 45.14 ml l^−1^ and agitation of 330 rpm.

## Discussion

Bioethanol production from the lignocellulosic waste materials depends on the treatment of the sawdust and removal of the hemicelluloses and lignin from the sawdust for the production of monosugars such as glucose. This is a challenge to the current researchers on the renewable fuel production (Meinita et al. 2012). Marine-derived *Trichoderma* is a highly potential source for the bioprospecting research (Saravanakumar and Kathiresan 2012). These marine *Trichoderma* strains were used in this work. The present study successfully attempted the pretreatment of the sawdust with 0.8 % phosphoric acid followed by enzyme hydrolysis using *H.estonica*-derived cellulase for the maximum production of monosugars and then conversion of bioethanol by fermentation with yeast (*S.cerevisiae*).

Statistically optimized conditions for the efficient sawdust hydrolysis process were pH (6.19), temperature (29 °C), cellulase enzyme (8.16 IU ml^−1^) and 8 % phosphoric acid-treated sawdust (7.95 mg l^−1^). For suitability of each pre-hydrolysis treatment for maximum solubilization of hemicelluloses and lignin, the effect of pre-hydrolysis treatment of sawdust with the enzyme was investigated using *H.estonica*-derived cellulase enzyme. It resulted in maximum yield of glucose (78.56 %). The significant conversion of the sawdust to the monosugars (glucose) was attained at temperature above 29 °C. This is due to the fact that high temperature increases hemicellulose degradation and lignin transformation, thus increasing the potential of cellulose hydrolysis (Li et al. 2009), whereas low temperature increases the solubilization of the hemicellulose (Sun and Cheng 2002). Addition of dilute acid in steam treatment can effectively improve enzymatic hydrolysis, decrease the production of inhibitory compounds, and lead to more removal of hemicelluloses (Martin et al. 2002; Kathiresan et al. 2011; Matsushita et al. 2010; Wang et al. 2009).

Saravanakumar and Kathiresan (2013a, b) have reported the potential of the marine strain over the terrestrial yeast strains and achieved the maximum yield of the bioethanol of 69.58 % under optimal conditions of temperature at 30 °C, sawdust concentration of 6.84 mg l^−1^ under the agitation speed of 360 rpm in 89 h of incubation. Similarly, the present study attained maximum bioethanol production of 85.6 % under the optimized conditions of temperature of 36.5 °C, incubation time of 102 h and enzyme-treated sawdust of 45.14 ml l^−1^ and agitation of 330 rpm; little variations of the optimized conditions were attributed due to the higher availability of monosugars in the *Trichoderma*-derived enzyme-treated sawdust hydrolysis. Thus, the temperature has a significant role in the bioethanol production by increasing the enzyme hydrolysis of the sawdust concentration and also the bioethanol yield due to increased production of fermentable monosugars (Fromanger et al. 2010; Zuroff and Curtis 2012). The present work suggested that the maximum production of the bioethanol from the sawdust could be achieved by proper method of acid and enzyme pretreatment and yeast fermentation employing *H.estonica* and *S.cerevisiae*.

## References

[CR1] Aro N, Pakula T, Penttiillae M (2005). Transcriptional regulation of plant cell wall degradation by filamentous fungi. FEMS Microbiol Rev.

[CR2] Askew DJ, Laing MD (1993). An adapted selective medium for the quantitative isolation of *Trichoderma* sp.. Plant Pathol.

[CR4] Fell JW (2005). The role of nucleotide analysis in the systematics of the yeast genera *Cryptococcus* sp. and *Rhodotorula* sp.. Stud Mycol.

[CR5] Fromanger R, Guillouet SE, Uribelarrea JL, Molina-Jouve C, Cameleyre X (2010). Effect of controlled oxygen limitation on *Candida shehatae* physiology for ethanol production from xylose and glucose. J Ind Microbiol Biotechnol.

[CR6] Kathiresan K, Saravanakumar K, Senthilraja P (2011). Bio-ethanol production by marine yeasts isolated from coastal mangrove sediment. Int Multidiscip Res J.

[CR7] Lau MJ, Lau MW, Gunawan C, Dale BE (2010). Ammonia fiber expansion (AFEX) pretreatment, enzymatic hydrolysis and fermentation on empty palm fruit bunch fiber (EPFBF) for cellulosic ethanol production. Appl Biochem Biotechnol.

[CR8] Li K (2003) The role of enzymes and mediators in lignocellulose degradation. In: Goodell B, Nicholas D, Schultz T (eds) American Chemical Society Symposium Series 845. Wood deterioration and preservation, advances in our changing world. American Chemical Society, Washington, DC, pp 196–209

[CR01] Li A, Antizar-Ladislao B, Khraisheh M (2007) Bioconversion of municipal solid waste to glucose for bioethanol production. Bioprocess Biosyst Eng 30(3):189–196 10.1007/s00449-007-0114-3 17458580

[CR9] Li XH, Yang HJ, Roy B, Wang D, Yue WF, Jiang LJ, Park EY, Miao YG (2009). The most stirring technology in future: cellulose enzyme and biomass utilization. Afr J Biotechnol.

[CR10] Martín C, Galbe M, Wahlbom CF, Hagerdal BH, Jonsson LJ (2002). Ethanol production from enzymatic hydrolysates of sugarcane bagasse using recombinant xylose**-**utilising *Saccharomyces cerevisiae*. Enz Microb Technol.

[CR11] Matsushita Y, Yamauchi K, Takabe K, Awano T, Yoshinaga A, Kato M, Kobayashi T, Asada T, Furujyo A, Fukushima K (2010). Enzymatic saccharification of Eucalyptus bark using hydrothermal pretreatment with carbon dioxide. Biores Technol.

[CR12] Mcmillan JD (1994) Fuels production In: Himmel ME, Baker JO, Overend RP (eds) ACS symposium series 566. American Chemical Society, Washington, DC, pp 292–324

[CR13] Meinita MD, Hong YK, Jeong GT (2012). Comparison of sulfuric and hydrochloric acids as catalysts in hydrolysis of *Kappaphycus alvarezii* (cottonii). Bioprocess Biosyst Eng Jan.

[CR14] Miller GL (1959). Use of DNS reagent for the measurement of reducing sugar. Anal Chem.

[CR15] Montgomery DC (2001). Design and analysis of experiments.

[CR16] Pakarinen A, Zhang J, Brock T, Maijala P, Viikari L (2011) Enzymatic accessibility of fiber hemp is from GH families 10 and 11 with thermostable cellulases in *lignocellulose* hydrolysis. 108(12):2823–2834

[CR17] Qureshi TA, Mirbahar KB, Samo MU, Soomro NM, Solangi AA, Memon A (2006). Clinical study of experimentally induced anaphylactic shock in goats. Int J Pharmacol.

[CR18] Rodriguez Gomez D, Lehmann L, Olkjar et al (2012) Examining the potential of plasma-assisted pretreated wheat straw for enzyme production by *Trichoderma**reesei.* Appl Biochem Biotechnol 166(8):2051–2063 (Data provider: Technical University of Denmark Document type) 10.1007/s12010-012-9631-x 22415783

[CR19] Sandhu H, Nidumolu U, Sandhu S (2012). Assessing risks and opportunities arising from ecosystem change in primary industries using ecosystem-based business risk analysis tool. Hum Ecol Risk Assess.

[CR20] Saravanakumar K, Kathiresan K (2012) Bioprospecting potential of marine-derived Trichoderma. Asian Pac J Trop Biomed 1**–**8

[CR21] Saravanakumar K, Kathiresan K (2013). Thermostable cellulase produced by trichoderma (*Hypocrea estonica*). J Biotechnol Sci.

[CR22] Saravanakumar K, Senthilraja P, Kathiresan K (2013b) Bioethanol production by mangrove derived marine yeast, *Sacchromyces cerevisiae* King Saud University Journal of King Saud University–Science 10.1016/j.jksus.2012.12.005

[CR23] Senthilraja P, Kathiresan K, Saravanakumar K (2011). Comparative analysis of bioethanol production by different strains of immobilized marine yeast. J Yeast Fungal Res.

[CR3] Sherrard EC, Harris EE (1932) Factors influencing properties of isolated wood lignin. Ind Eng Chem 24:103

[CR24] Sun Y, Cheng J (2002). Hydrolysis of lignocellulosic material from ethanol production: a review. Bioresour Technol.

[CR25] Sveinsdottir M, Baldursson SRB, Orlygsson J (2009). Ethanol production from monosugars and lignocellulosic biomass by thermophilic bacteria isolated from Icelandic hot springs. Icelandic Agric Sci.

[CR26] Wang Y, Sakamoto Y, Kamiya Y (2009). Remediation of actual groundwater polluted with nitrate by the catalytic reduction over copper-palladium supported on active carbon. Appl Catal A.

[CR27] Wingren A, Galbe M, Zacchi G (2003). Techno economic evaluation of producing ethanol from softwood: comparison of SSr and SHF and identification of bottlenecks. Biotechnol Prog.

[CR28] Zuroff TR, Curtis WR (2012). Development of symbiotic consortia for *lignocellulosic* biofuel production. Appl Microbiol Biotechnol.

